# The bridge between design and analysis

**DOI:** 10.1007/s40037-017-0367-8

**Published:** 2017-07-14

**Authors:** Jimmie Leppink, Patricia O’Sullivan, Kal Winston

**Affiliations:** 1 Maastricht University, Maastricht, The Netherlands; 2 University of California, San Francisco, USA; 3 The Commonwealth Medical College, Scranton, PA USA

## Abstract

The overall purpose of the ‘Statistical Points and Pitfalls’ series is to help readers and researchers alike increase awareness of how to use statistics and why/how we fall into inappropriate choices or interpretations. We hope to help readers understand common misconceptions and give clear guidance on how to avoid common pitfalls by offering simple tips to improve your reporting of quantitative research findings. Each entry discusses a commonly encountered inappropriate practice and alternatives from a pragmatic perspective with minimal mathematics involved. We encourage readers to share comments on or suggestions for this section on Twitter, using the hashtag: #mededstats

Using tools for statistical analysis that do not match with the design of the study increases the chance that conclusions drawn from that analysis are incorrect. Through a concise example of how failing to account for study design characteristics in the statistical analysis can result in incorrect conclusions with regard to specific comparisons of interest, this entry illustrates that key characteristics of the study design should drive choices at the stage of analysis.

## Example study

One area of study in educational research compares learning from examples with learning by solving problems [1, 2]. A main research question in this area of study is whether students learn more from solving problems, from studying worked examples or from some combination thereof. Suppose that some researchers randomly assign *N* = 140 medical students to four conditions (*n* = 35 participants per condition): problem-problem, problem-example, example-problem, example-example. As illustrated in Table 1, the design of this study is a so-called *two-way design*: first task (problem/example) and second task (problem/example). In other words, first and second task constitute two factors in a 2 by 2 *factorial design* [1, 3].

**Table 1:** Design of the example study: 2 by 2 (i. e., two-way) factorial

		Factor 2:	Second task
		*Problem*	*Example*
Factor 1: First task	*Problem*	*n* = 35 participants	*n* = 35 participants
	*Example*	*n* = 35 participants	*n* = 35 participants

Participants in the problem-problem condition try to solve two problems – problem A and problem B – that follow the same structure and are of similar difficulty. In the problem-example condition, participants first try to solve problem A and then study a worked example of problem B. In the example-problem condition, participants first study a worked example of problem A and then try to solve problem B. Finally, in the example-example condition, participants study worked examples of both problems and solve none of the problems by themselves. Subsequently, participants in all four conditions complete the same post-test, which comprises ten problems of the same structure as problems A and B and are of similar difficulty. Each post-test problem is scored ‘0’ whenever a participant provides an incorrect solution and ‘1’ when that participant provides a correct solution. Hence, a participant’s total score on the post-test can range from 0 to 10. The researchers find an average score of 4.79 (*SD* = 0.96) in the problem-problem condition, 5.07 (*SD* = 1.05) in the problem-example condition, 5.20 (*SD* = 1.04) in the example-problem condition, and 5.42 (*SD* = 0.96) in the example-example condition. The findings from this simulated example study are similar to those from an actual experiment with these conditions published fairly recently [1].

## Commonly encountered analytic approaches in the example study

Broadly speaking, researchers might consider three analytic approaches for the example study: (1) a statistical test (i. e., *t*-test) for the difference in average score for each pair of conditions; (2) one overall statistical test across the four conditions (i. e., one-way analysis of variance, ANOVA [4]); and (3) a two-way ANOVA in which three statistical tests are performed: the effect of first task, the effect of second task, and their combined effect. As outlined in the following, the first two approaches incorrectly treat the data as from a one-way design: ‘first-and-only task’ with four possibilities (e. g., method A, method B, method C or method D). Consequently, these approaches fail to address the question with regard to the effect of first task, the effect of second task, and their combined effect. The third approach, two-way ANOVA, is the only approach that correctly treats the data as two-way and is therefore the only appropriate approach for this type of data [1, 3].

Researchers who follow the first approach perform a *t*-test for each pair of conditions. Given *k* conditions, there are [*k* × (*k* − 1)]/2 pairs of conditions. Hence, three conditions (*k* = 3) yields three pairs (i. e., 1‑2, 1‑3, 2‑3) and four conditions (*k* = 4) yields six pairs (i. e., 1‑2, 1‑3, 1‑4, 2‑3, 2‑4, 3‑4). Thus, in the example study, this approach comes down to six *t*-tests in total, more than is needed for the type of design in this study [1, 5]. Performing more statistical tests than is needed tends to elevate the number of incorrect rejections of null hypotheses (i. e., *Type I errors*). To understand the latter, consider the following example. A fair die has six options – ‘1’, ‘2’, ‘3’, ‘4’, ‘5’, and ‘6’ – that each have the same chance of occurring. Hence, if we throw one die, the chance of obtaining ‘6’ is 1/6 or nearly 0.17. However, if we throw two dice, there are 6 × 6 = 36 possible combinations of options, 11 of which yield a ‘6’ at least once: ‘16’, ‘26’, ‘36’, ’46, ‘56’, ‘66’, ‘65’, ‘64’, ‘63’, ‘62’, and ‘61’. In the same way as each option has the same chance of occurring with one die, all the combinations of two dice also have the same chance of occurring.

Hence, the chance of obtaining ‘6’ at least once when throwing two dice is as large as 11/36 ≈ 0.31. Increasing the number of dice, the chance of obtaining ‘6’ at least once increases further. This reasoning also applies to statistical testing. A statistical significance test is like the event of throwing ‘6’ but with a lower chance, since the statistical significance level is usually 0.05 not 1/6. With one test, the chance of rejecting a true null hypothesis is 5%; with two tests the chance of rejecting at least one true null hypothesis is almost 10%, and this chance increases further in the case of more tests.

Researchers who follow the second approach perform a one-way ANOVA to test for any differences between the four conditions. If that overall test yields a statistically significant outcome, they follow up with a *post-hoc* testing procedure in which *t*-tests for all or a selected number of pairs of conditions are carried out at a lower statistical significance level to keep Type I error probability limited [4]. Performing one-way ANOVA on the reported findings in the example study, we find *p* = 0.073. Since this outcome is not statistically significant at the conventional 0.05 significance level, there is no reason to follow up with the aforementioned post-hoc testing procedure. Although in this second approach the chance of a Type I error is lower than in the first approach, both approaches fail to address the questions with regard to the effect of first task, the effect of second task, and their combined effect (cf. Table 1), and are therefore inappropriate for this type of data (i. e., two-way data) [1, 3].

**Fig. 1:**
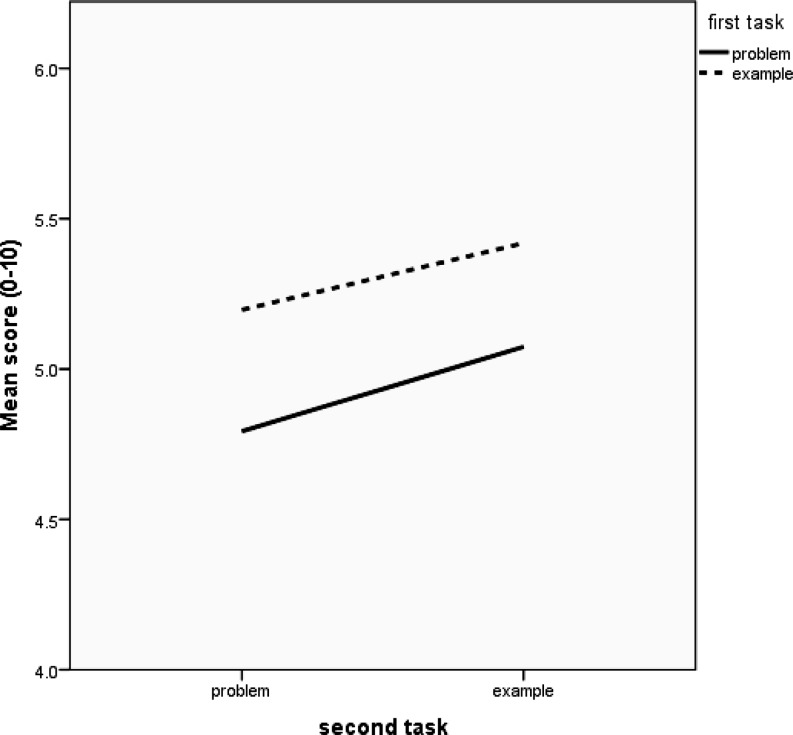
Graphical representation of the average scores of the four conditions in two-way analysis

Some researchers acknowledge that the design of the example study is a two-way design. Fig. 1 correctly represents the four conditions as 2 by 2 in a two-way design (cf. Table 1).

Given that this third approach is the correct one, we focus on this approach in the remainder of this entry.

## Different types of effects

Fig. 1 indicates that first and second task have so-called *additive effects *or main effects [4] on post-test score: the lines in the graph are more or less parallel. Participants who started with an example on average performed a bit better on the post-test than their peers whose first task was to solve the problem by themselves (i. e., main effect of first task). Additionally, participants whose second task was to study an example performed better than their peers who had to solve the problem by themselves (both lines are sloping upwards). The more or less parallel lines indicate that the beneficial effect of the first task being an example (i. e., the effect of the first task) is the same regardless of whether the second task is a problem or an example. Likewise, the beneficial effect of the second task being an example (i. e., the effect of the second task) is not moderated by what participants were asked to do in the first task.

**Fig. 2:**
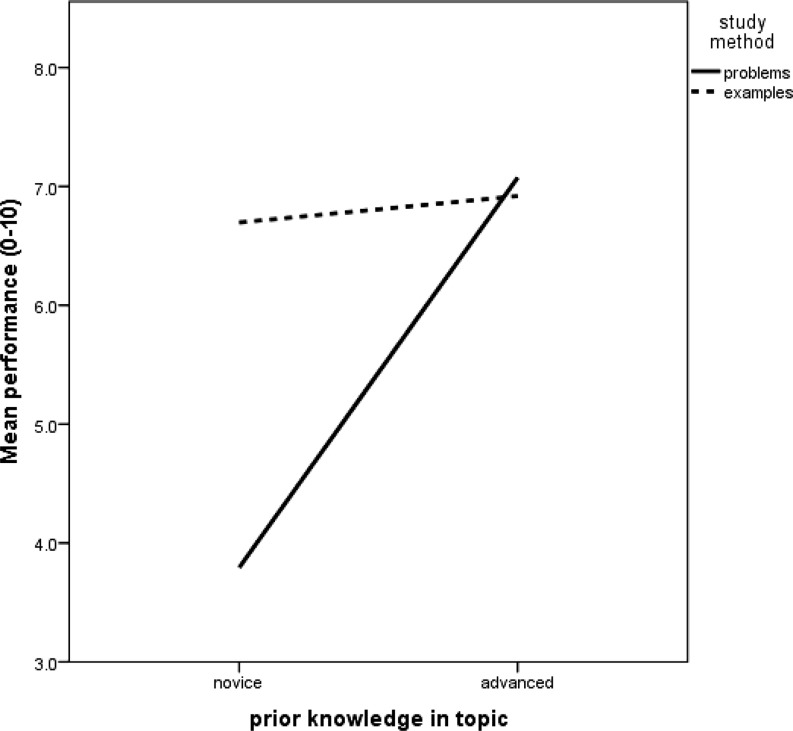
Example of an interaction effect: the effect of study method (i. e., solving problems vs. studying worked examples) depends on the type of learner

If the lines in Fig. 1 had gone in clearly different directions (e. g., crossing lines), this would have indicated a so-called *combined effect *or *interaction effect* of first and second task. In that case, the effect of the first task would be different for participants whose second task was an example than for participants whose second task was to solve a problem. Likewise, the effect of the second task would then be different for participants who started with an example than for participants who started with a problem. A practical example of such an interaction effect is the so-called *expertise reversal effect* [6]: instructional support (e. g., studying a worked example) that is beneficial for novice learners is not effective or even negatively affects learning among more advanced learners. Fig. 2 demonstrates an example of this phenomenon.

To distinguish between interaction and main effects, we need to represent the four conditions as 2 by 2 as in Table 1 and Fig. 1 and 2. Performing two-way ANOVA, we obtain three tests, as displayed in Table 2.

**Table 2:** Outcomes of two-way ANOVA: *p* -values, 95% confidence intervals (CI), and Bayes factors for the alternative vs. the null (*BF*_*10*_) and for the null vs. the alternative hypothesis (*BF*_*01*_)

Effect	*p*-value	95% CI ^a^		*BF* ^b^	
		Lower bound	Upper bound	*BF*_10_	*BF*_01_
First task	0.029	0.039	0.709	1.649	0.606
Second task	0.140	−0.084	0.586	0.484	2.066
First-by-second	0.862	−0.729	0.611	0.192	5.209

^a^ 95% CI of the difference: positive values indicate favour of example over problem

^b^ *BF*_01_ = 1/*BF*_10_

Using *p* -values and testing at the conventional 0.05 significance level, we see that only the main effect of first task is statistically significant (*p* = 0.029). This information is also provided by the 95% confidence intervals [7]: the interval for the main effect of first task is the only one that does not include zero. Using Bayes factors, which quantify the strength of evidence against vs. in favour of a null hypothesis (*H*_0_) [8, 9], we see that the only Bayes factor that indicates a preference towards the alternative hypothesis (*H*_1_: there is an effect) vs. the null hypothesis (*H*_0_: there is no effect) is that for the main effect of first task, because the Bayes factor for *H*_1_ vs. *H*_0_ (*BF*_10_) is larger than 1 (i. e., 1.649). This Bayes factor indicates some, though weak (i. e., *BF* < 3.2), evidence in favour of *H*_1_ [9]. For the main effect of second task, we find weak evidence in favour of the null hypothesis (*BF*_01_ = 2.066). For the interaction effect, we find substantial evidence (i. e., 3.2 < *BF* < 10 [9]) in favour of the null hypothesis (*BF*_01_ = 5.209). To conclude, with regard to the effects of first task, second task, and their combined effect (cf. Table 1), it seems that what matters most, if anything, is that the first task is an example rather than a problem.

## Maximising the probability of detecting effects of interest

Apart from the fact that two-way analysis correctly accounts for the study design, it is also more likely than the other two previously discussed approaches to detect effects of interest. Using G*Power [10], a program for statistical power and required sample size calculations, we learn that a *t*-test for the difference in average post-test score between two conditions of *n* = 35 each has a statistical power of about 0.54 using a significance level of 0.05 and assuming a medium size (i. e., half a standard deviation) difference between conditions. In other words, in about half of the tests we would fail to detect a real difference (i. e., *Type II error*). By comparison, a one-way ANOVA, under the given circumstances, has a statistical power of about 0.68 meaning that one of every three tests would fail to detect a real difference. In fact, in the example study, the outcome of one-way ANOVA is not statistically significant. Finally, two-way ANOVA in this case has a statistical power of about 0.84 meaning that only about one of every six tests would fail to detect a real difference.

The difference in statistical power can be explained in an intuitive manner as follows. Keeping other factors the same, statistical power increases with sample size. In the example study, every pairwise *t*-test involves a comparison of two conditions of *n* = 35 each, hence a sample of 70 in total. Although the one-way ANOVA does include the full sample of *N* = 140, the conditions compared are still of size *n* = 35; the question answered by one-way ANOVA is whether there is ‘any difference’ between the four conditions of *n* = 35 each. In two-way ANOVA, each test involves a comparison of two groups vs. two other groups. The test on the main effect of the first task pertains to the difference of starting with a problem (i. e., problem-problem *or* problem-example: *n* = 35 + 35 = 70) vs. starting with an example (i. e., example-problem *or* example-example: *n* = 35 + 35 = 70). The test on the main effect of the second task is about the difference of the second task being a problem (i. e., problem-problem *or* example-problem: *n* = 35 + 35 = 70) vs. the second task being an example (i. e., problem-example *or* example-example: *n* = 35 + 35 = 70). Finally, the interaction effect involves the third possible contrast: problem-problem or example-example (*n* = 35 + 35 = 70) vs. problem-example or example-problem (*n* = 35 + 35 = 70). Thus, with two-way ANOVA, the conditions compared are of size *n* = 70.

## When separate tests make sense and when they do not

We have provided two reasons for favouring two-way ANOVA over both *t*-tests and one-way ANOVA when analysing data from a two-way design: accounting for the characteristics of the study design and increasing statistical power. However, in the two-way ANOVA approach, there is one situation when following up with specific *t*-tests tends to make sense and that is when we have sufficient grounds to reject *H*_0_ of ‘no interaction’ [3, 5]. After all, an interaction effect dictates that the effect of one factor depends on the second factor. Had there been differences such that the lines were non-parallel (e. g., had the pattern in Fig. 1 been that of Fig. 2), one could perform a *t*-test for the difference between problem-problem and example-problem and another *t*-test for the difference between problem-example and example-example. Note, however, that we are using *t*-tests only as a follow up on a significant interaction effect and that we are doing two specific and not all the possible (i. e., six) *t*-tests.

## To conclude

Researchers should bear in mind a bridge between design and analysis, such that study design characteristics drive analytic choices and the analysis appropriately accounts for the characteristics of the study design. If we perform one-way analysis of two-way data, through pairwise *t*-tests or one-way ANOVA, we fail to address questions with regard to the three contrasts that matter in a two-way design: two main effects and their interaction effect. Performing two-way ANOVA, we directly test these three contrasts. Consequently, compared to the pairwise *t*-tests approach, we keep the chance of a Type I error limited by performing three contrast tests instead of six pairwise *t*-tests. Simultaneously, compared with both the pairwise *t*-tests and one-way ANOVA approach, two-way ANOVA comes with a lower chance of Type II error (i. e., increased statistical power) because the three contrast tests maximize the sample size for each test.
